# 3D Imaging Evaluation of Distal Reference Axes for Tibial Rotational Alignment in Medial-Type Knee Osteoarthritis

**DOI:** 10.7759/cureus.97381

**Published:** 2025-11-20

**Authors:** Taro Uehara, Takaaki Shishido, Ishida Tsunehito, Toshinori Masaoka, Tateiwa Toshiyuki, Kyohei Nagayama, Yasuhito Takahashi, Kengo Yamamoto

**Affiliations:** 1 Orthopaedic Surgery, Tokyo Medical University, Tokyo, JPN; 2 Orthopaedic Surgery, Tokyo Medical University Hospital, Tokyo, JPN

**Keywords:** akagi's line, distal reference axis, femoro-tibial angle, kellgren-lawrence grade, knee osteo-arthritis, rotational alignment, tka (total knee arthoplasty)

## Abstract

Background

Precise tibial rotational alignment is essential for successful total knee arthroplasty (TKA). While Akagi’s line is established proximally, the optimal distal reference axis remains uncertain.

Methods

We analyzed 120 medial knee osteoarthritis (OA) patients (mean age 71.7 years) using three-dimensional computed tomography. The proximal anteroposterior (AP) axis (Akagi’s line) was compared with three distal axes: ankle AP (d-AP1), talar AP (d-AP2), and second metatarsal bone axis (d-AP3). Associations with Kellgren-Lawrence grade and femorotibial angle were assessed.

Results

Mean deviations from the proximal axis were -17.55 ± 9.49° (d-AP1), -3.36 ± 10.13° (d-AP2), and -0.85 ± 11.18° (d-AP3). d-AP3 showed the closest alignment and was unaffected by OA severity, though with gender-related variation. d-AP1 rotated externally with increasing varus deformity.

Conclusion

The second metatarsal axis (d-AP3) provides the most reliable distal reference for tibial rotational alignment in TKA, particularly when proximal landmarks are inaccessible during minimally invasive surgery.

## Introduction

To achieve stable postoperative outcomes of total knee arthroplasty (TKA) in patients with knee osteoarthritis (OA), accurate placement with proper alignment of each component is important [1,2]. Accurate rotational installation of the tibial component in the transverse plane is a crucial element of the TKA procedure, as inaccurate rotational placement may lead to inadequate rotational conformity of the tibiofemoral joint, problems of patellar tracking, excessive or insufficient coverage of the transverse plane of the tibia by the tibial component, and abnormal toe direction [3,4].

The proximal anteroposterior (AP) axis of the tibia is used as an intraoperative indicator to achieve proper tibial rotational alignment. To date, various proximal AP axes of the tibia have been reported as possible indicators [2,5-7]. Among these, the line connecting the middle of the posterior cruciate ligament and the medial border of the patellar tendon attachment (Akagi’s line) is now widely used because it is highly consistent with the AP axis of the femur in the normal knee [6,8-10].

However, recent minimally invasive surgical techniques for TKA rarely extend to the patellar attachment area of the tibial tuberosity. In addition, many patients show ankle and foot deformities with worsening of their knee OA, and hence, determining the rotational alignment of the lower leg based solely on the proximal AP axis may not be reliable. Therefore, it is important to intraoperatively confirm the rotational alignment determined using the proximal AP reference axis with the distal AP reference axis.

Multiple axes have been proposed as useful distal reference axes of the lower limb, based on their relationship with the proximal AP reference axis. However, there have been few reports to date regarding the usefulness of the distal AP axes for the determination of rotational alignment, and it has been reported that the proximal and distal AP axes may be separated depending on the severity of the OA deformation and various characteristics of the patients [11-16].

If a distal AP axis could be identified as a stable and reliable reference, analogous to Akagi’s line for the proximal tibia, it would enable accurate rotational alignment of the tibial component regardless of deformity severity or surgical exposure. Such a reference could potentially reduce malrotation-related complications and improve clinical outcomes following TKA.

The purpose of this study was to identify the most appropriate reference axis among the three representative distal AP axes for use in medial knee OA, by detailed evaluation using three-dimensional computed tomography (3DCT), and to clarify the effects of OA progression on the alignment of the proximal and distal AP axes.

## Materials and methods

Study design

This study was designed as a retrospective observational analysis conducted at the authors’ institution. All cases involved patients who underwent primary TKA for medial knee OA between January 2020 and April 2022. The protocol was reviewed and approved by the institutional review board, and written informed consent was obtained from all participants prior to enrollment.

Patient recruitment

A total of 169 consecutive patients (67 men, 102 women) who underwent primary TKA at our institution between January 2020 and April 2022 were included. They were informed of the risk of radiographic exposure from the CT scans and radiographs, and written informed consent was obtained. They were treated during the specified study period and fulfilled the criteria for inclusion in the present investigation.

Inclusion and exclusion criteria

**Figure 1:**
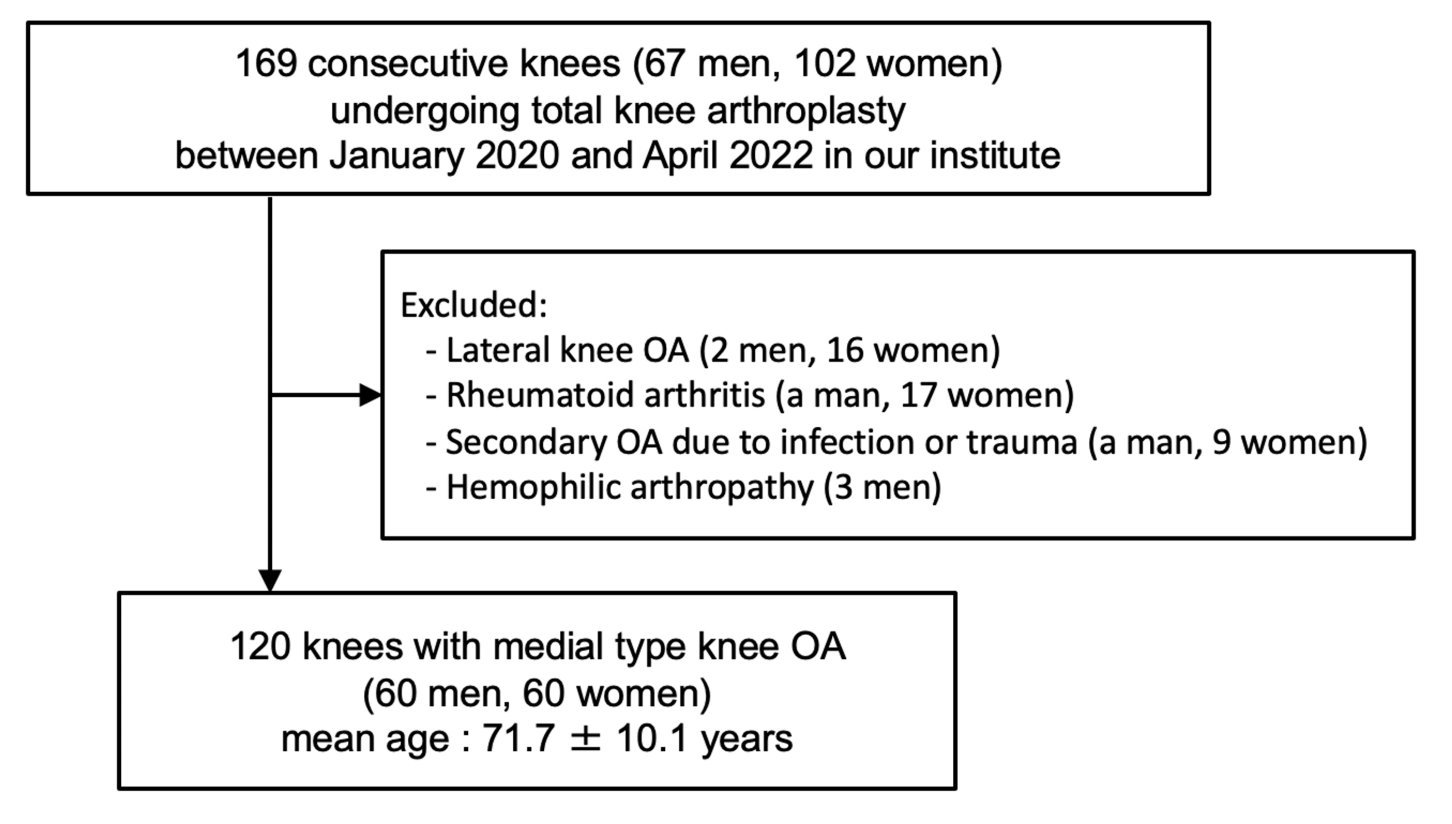
Flowchart of patient recruitment and selection Flow diagram showing the inclusion and exclusion process of patients with medial knee osteoarthritis (OA) who underwent primary total knee arthroplasty (TKA) between January 2020 and April 2022. A total of 120 consecutive knees (60 men and 60 women; mean age, 71.7 ± 10.1 years) were included after excluding cases with lateral OA, rheumatoid arthritis, secondary OA due to infection or trauma, and hemophilic arthropathy.

Eligibility was restricted to patients diagnosed with medial knee OA who underwent primary TKA at our institution. Patients were excluded if they had lateral knee OA (two men and 16 women), rheumatoid arthritis (one man and 17 women), secondary OA attributable to pyogenic or post-traumatic arthritis (one man and nine women), and hemophilic joint disease (three men). Finally, a total of 120 patients (60 men and 60 women) with medial knee OA (cases with a femorotibial angle (FTA) of 178 degrees or greater) were included in the cohort (Figure 1). This approach ensured a relatively homogeneous study population by eliminating cases with confounding etiologies of knee joint degeneration.

Data collection

**Figure 2:**
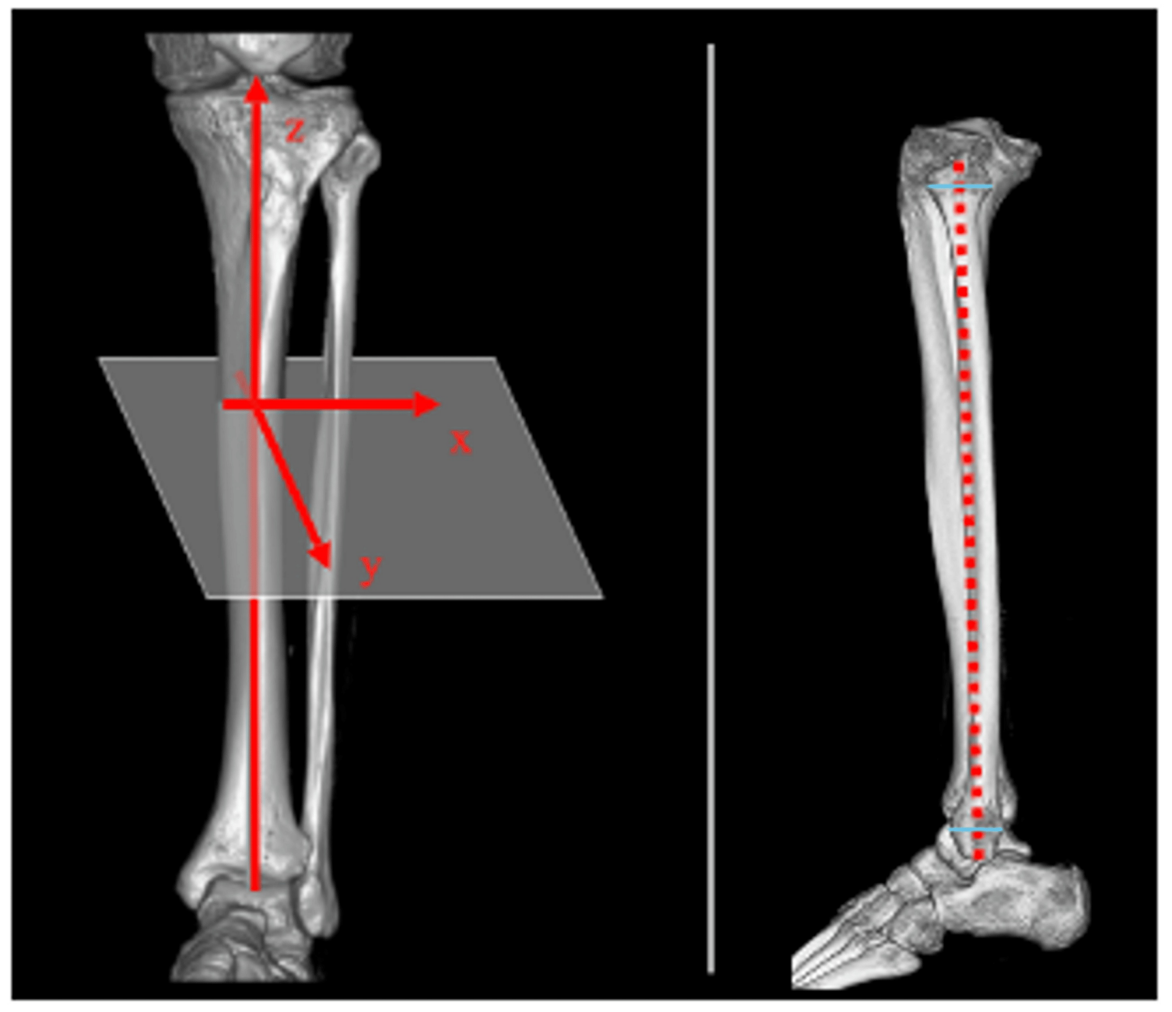
Definition of the 3D coordinate system In the 3D-reconstruction images, the tibial functional bone axis was defined as the sagittal axis (z-axis). The red dotted line indicates the fibular axis.

CT imaging data and standing single longitudinal radiographs of the whole lower extremities that were taken for preoperative planning were analyzed. CT scans of the patients’ knees were taken at levels ranging from 40 mm proximal to the knee joint to the toe using a LightSpeed VCT CT scanner (GE Healthcare Ltd, Milwaukee, WI). During the scanning, the patient’s leg was fixed in a carbon frame so that the lower limb was maintained with the knee extended and in the mid-ankle position as much as possible. The slice thickness of the CT scan was 1.25 mm. CT images were reconstructed in 3D using GE Advantage Workstation AW 4.4 software (GE Healthcare Ltd, Milwaukee, WI). This workstation was also used for drawing and projecting lines or points on 3D images, and for measuring the angles between two lines. In the 3D reconstruction, the functional axis of the tibia, which is the line passing through the anterior edge of the anterior cruciate ligament attachment on the tibial plateau and parallel to the fibular shaft axis, was defined as the sagittal axis (z-axis). The fibular shaft axis was defined as the line connecting midpoints of the outer cortical diameter of the proximal and distal ends of the fibular diaphysis (Figure 2) [17]. 

**Figure 3:**
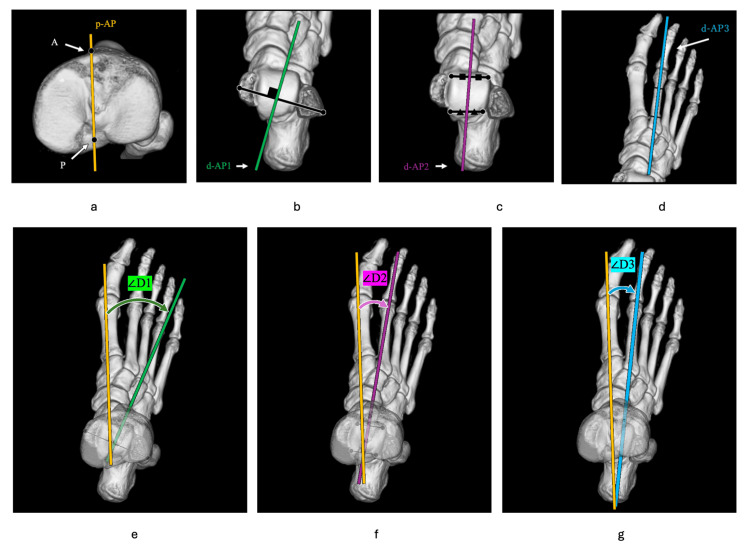
Definition of axes and angles on 3D reconstructed CT a) The p-AP axis was defined as the line connecting the medial border of the tibial tuberosity (A) and the center of the posterior cruciate ligament attachment (P). b) The d-AP1 was defined as the AP axis of the ankle joint. c) The d-AP2 was defined as the AP axis of the talus. d) The d-AP3 was defined as the second metatarsal bone axis. e, f, g) The angles of the d-AP1 (e), d-AP2 (f), and d-AP3 (g) to the p-AP were defined as ∠D1, ∠D2, and ∠D3, respectively. All measurements were performed with an external rotation direction of +.

The proximal AP axis of the tibia (p-AP) was defined as the line connecting the medial border of the tibial tuberosity (A) and the center of the posterior cruciate ligament (PCL) attachment (P) (Figure 3a). P was defined as the point at the center of the posterior fossa in the coronal CT sections, and at the superior border of the PCL attachment area in the sagittal CT sections. Three distal tibial AP axes, namely, d-AP1, d-AP2, and d-AP3, were defined on the vertical view of the 3DCT images. The d-AP1 was defined as the AP axis of the ankle joint; the most prominent points of the medial and lateral malleolus were identified on axial CT slices in the region where the malleolus contours were maximal. The transmalleolar axis was drawn by connecting these two points, and d-AP1 was defined as the line perpendicular to this axis in the same axial plane (Figure 3b). The d-AP2 was defined as the AP axis of the talus; the anterior and posterior articular margins of the talar dome at the tibiotalar joint surface were identified on sagittal and axial reconstructed images. The midpoints of these margins were marked and connected to form the AP axis of the talus (Figure 3c). The d-AP3 was defined as the second metatarsal axis, formed by connecting the midpoints of the upper and lower quarter points along the entire length of the second metatarsal on the 3D reconstructed image (Figure 3d). The angles of the d-AP1, d-AP2, and d-AP3 to the p-AP were defined as ∠D1, ∠D2, and ∠D3, respectively. Measurements were performed using the 3D-reconstructed images looking down on the lower leg. Angular measurements were taken by projecting the p-AP and the d-AP axes onto a plane perpendicular to the z-axis. All measurements were performed with an external rotation direction of + (Figure 3e-3g).

Definition of axes and angles on 3D reconstructed CT

Two independent observers (TU and TY) who were not involved in the surgery measured each angle twice at intervals of one week or more, and the mean values were used for analysis. Furthermore, all measurements were performed while blinding the patient’s background information and radiological evaluation results. Intraobserver variations were assessed by a single observer (TU). The three angular measurements described above were repeated 10 times in three patients chosen arbitrarily. The maximum intraobserver difference between measurements was 2°, and the largest standard deviation was 0.8°, which was observed in the measurement of ∠D2. The maximum interobserver differences in the measurements of ∠D1, ∠D2, and ∠D3 were 2°, 3°, and 1°, respectively. The interobserver intraclass correlation coefficient (ICC) values between the two observers (TU and TY) were 0.988 (95% CI, 0.982-0.991), 0.989 (95% CI, 0.984-0.992), and 0.998 (95% CI, 0.998-0.999) for ∠D1, ∠D2, and ∠D3, respectively, indicating excellent reliability. Intraobserver ICCs were also excellent: 0.996 (95% CI, 0.984-1.000) for ∠D1, 0.993 (95% CI, 0.970-1.000) for ∠D2, and 0.945 (95% CI, 0.798-0.999) for ∠D3. The progression of knee OA was assessed using the Kellgren-Lawrence (KL) classification [18], and FTA was measured from the standing whole lower extremity plain radiographs. Knee joints were divided into three groups, namely, KL-II, KL-III, and KL-IV. The KL classification of each patient was determined by a single proficient observer (TU). FTA was measured twice each by two observers (TU and KN). The mean of all measurements was regarded as the true value. The maximum intraobserver difference between measurements of FTA was 1°, and the maximum interobserver difference between measurements was 1°. Whether there was an association between the angle of the d-AP axes to the p-AP axis and progression of knee OA (FTA and KL classification) was investigated.

Statistical analysis

A one-sample t-test against a null hypothesis of zero mean was conducted for each of the angles ∠D1, ∠D2, and ∠D3. The t-test was used to compare each measured value between men and women. One-way ANOVA was used for statistical analysis between the angle of the d-AP axes to the p-AP axis and the KL classification. The r and Cramer's V were reported as effect size indices for the t-test and one-way ANOVA, respectively. r and V = 0.1 are considered to represent a small effect, r and V = 0.3 a moderate effect, and r and V = 0.5 a large effect. The Pearson correlation coefficient was used for statistical analysis between the angle of the d-AP axes to the p-AP axis and FTA. Analyses were conducted using the SPSS software package (SPSS, version 17, Chicago, IL. A p-value of less than 0.05 was considered to indicate a statistically significant difference between groups.

## Results

Patient demographics

A total of 120 knee joints from 120 patients were analyzed, comprising 60 men and 60 women with a mean age of 71.7 ± 10.1 years. The mean FTA was 183.3 ± 4.2°. Based on the KL classification, 11 knees were grade II, 32 knees were grade III, and 77 knees were grade IV (Table 1).

**Table 1:** Patient demographics Values are presented as numbers unless otherwise indicated. *Age and FTA are shown as the mean ± standard deviation. FTA, femorotibial angle; KL, Kellgren-Lawrence classification

Demographic	Value
Number of knee joints	120
Gender (males:females)	60:60
Age* (year)	71.7 ± 10.1
FTA* (degree)	183.3±4.2
KL classification	
KL-Ⅱ	11
KL-Ⅲ	32
KL-Ⅳ	77

Measurement of the angle of the distal to proximal AP axes of the lower limb

In all patients, the measurements of ∠D1, ∠D2, and ∠D3 were as follows: ∠D1 (angle of the d-AP1; the AP axis of the ankle joint to the p-AP axis): mean (SD) = -17.55 (9.49), 95% CI (-19.08, -16.01), d = -2.07, p < 0.001; ∠D2 (angle of the d-AP2; the AP axis of the talus to the p-AP axis): mean (SD) = -3.36 (10.13), 95% CI (-5.19, -1.52), d = -0.33, p < 0.001; and ∠D3 (angle of the d-AP3; the second metatarsal bone axis to the p-AP axis): mean (SD) = -0.85 (11.18), 95% CI (-2.87, 1.17), d = -0.08, p = 0.406. Among the 3 d-AP axes, the d-AP3 axis showed the greatest mean consistency with the p-AP axis, whereas it also demonstrated the largest intercase variability. Regarding sex differences, there was no significant difference between men and women in ∠D1 (p = 0.16). On the other hand, ∠D2 and ∠D3 were both significantly smaller in women than in men (p = 0.006, d = 0.52 and p = 0.05, d = 0.35 respectively), indicating moderate to large effect sizes. In other words, the d-AP2 and the d-AP3 tended to be more internally rotated relative to the d-AP in women than in men (Table 2).

**Table 2:** Differences in ∠D1, ∠D2, and ∠D3 by patient sex Comparison of the mean angles of ∠D1, ∠D2, and ∠D3 between male and female patients. Values are presented as the mean ± standard deviation (degrees). p-values were calculated using the one-sample t-test, with the null hypothesis set at zero.

	Men (n = 60)	Women (n = 60)	p-value	Effect size
∠D1 (degrees)	-18.6 ± 9.0	-16.3 ± 8.1	0.16	n/a
∠D2 (degrees)	-0.8 ± 11.5	-5.9 ± 7.8	0.006	0.52
∠D3 (degrees)	1.3 ± 10.6	-2.6 ± 11.7	0.05	0.35

Association between torsion of the lower limb and progression of knee OA

**Figure 4:**
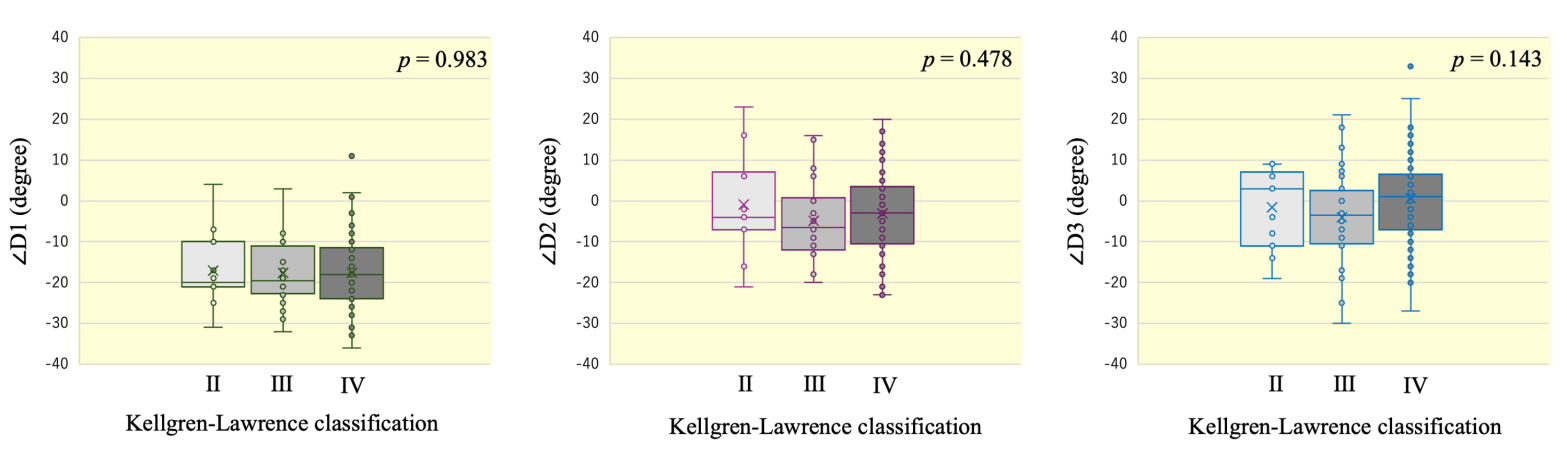
Box-and-whisker plots showing the distributions of ∠D1, ∠D2, and ∠D3 in knee joints of KL grades II, III, and IV Boxes represent the interquartile range (IQR), horizontal lines indicate the median, and crosses indicate the mean. Whiskers extend to 1.5 × IQR, and outliers are plotted as individual points. No significant differences in the angles were observed among the three KL groups.

Based on the KL classification, 11 knee joints were classified as group II, 32 knee joints as group III, and 77 knee joints as group IV. The results of one-way ANOVA to compare each mean angle of the d-AP axes to the p-AP axis among the 3 KL-grade groups were as follows: ∠D1: F(2, 117) = 0.017, p = 0.983; ∠D2: F(2, 117) = 0.743, p = 0.478; and ∠D3: F(2, 117) = 1.976, p = 0.143. These results indicated that there were no significant differences among the 3 KL-grade groups (Figure 4).

The same analysis was performed separately for sex, and similarly, no significant differences were found among the KL-grade groups (Table 3).

**Table 3:** Comparison of the distal AP axes relative to the proximal AP axis in patients grouped by KL grade Comparison of the mean angles of the three distal AP axes (∠D1, ∠D2, and ∠D3) relative to the proximal AP axis among the 3 KL-grade groups (KL-II, KL-III, and KL-IV). Values are presented as the mean ± standard deviation (degrees). p-values were calculated using one-way ANOVA.

		KL-Ⅱ (n = 11)	KL-Ⅲ (n = 32)	KL-Ⅳ (n = 77)	p-value
∠D1 (degrees)	Total	-17.1 ± 9.1	-17.6 ± 7.5	-17.6 ± 8.7	0.983
	Men	-20.7 ± 7.2	-20.1 ± 5.3	-18.4 ± 9.3	0.860
	Women	-13.5 ± 10.2	-16.8 ± 7.9	-16.5 ± 7.7	0.946
∠D2 (degrees)	Total	-0.9 ± 12.4	-4.9 ± 8.5	-3.0 ± 10.2	0.478
	Men	5.7 ± 12.5	-2.0 ± 8.9	-1.4 ± 11.6	0.462
	Women	-10.0 ± 6.4	-5.9 ± 8.2	5.3 ± 7.4	0.533
∠D3 (degrees)	Total	-1.6 ± 9.5	-4.0 ± 11.5	0.6 ± 10.9	0.143
	Men	4.0 ± 6.9	0.7 ± 6.0	1.0 ± 11.6	0.513
	Women	-11.3 ± 5.7	-5.6 ± 12.4	0.0 ± 9.8	0.867

**Figure 5:**
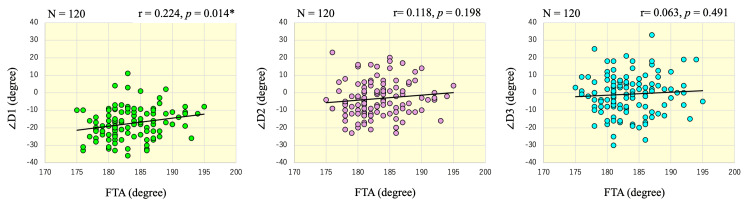
Scatter plot showing the correlations of ∠D1, ∠D2, and ∠D3 with FTA Scatter plots illustrating the relationships between FTA (x-axis) and ∠D1, ∠D2, and ∠D3 (y-axis). Each plot includes 120 knees, with regression lines shown. A positive correlation was found for ∠D1, while ∠D2 and ∠D3 showed no significant associations with FTA.

The mean FTA of all knee joints was 183.3 ± 4.2°. The Pearson correlation coefficient showed a modest positive correlation between FTA and ∠D1 (r = 0.224, p = 0.014). In other words, the AP axis of the ankle was found to be slightly externally rotated with progression of the varus deformity of the knee. There was no significant correlation between FTA and ∠D2 (r = 0.118, p = 0.198) and ∠D3 (r = 0.063, p = 0.491) (Figure 5).

**Figure 6:**
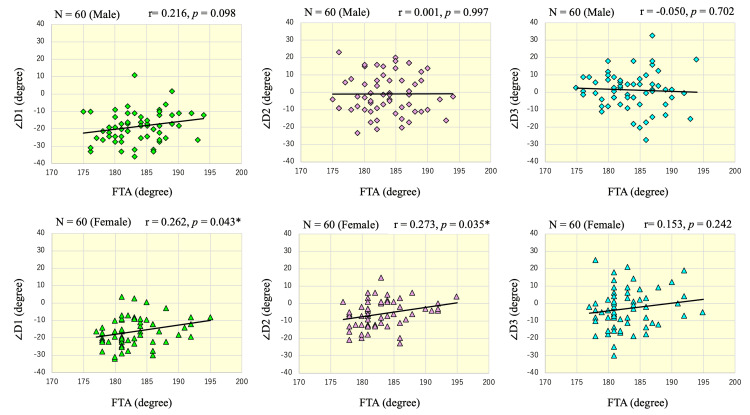
Scatter plots showing the association between ∠D1, ∠D2, or ∠D3 and FTA, stratified by sex Scatter plots illustrating the relationships between FTA (x-axis) and ∠D1, ∠D2, and ∠D3 (y-axis). Each plot includes 60 knees, with regression lines shown. In men (top row), no significant correlations were found for any combination, whereas in women (bottom row), significant positive correlations were observed between ∠D1 and FTA (r = 0.262, p = 0.043), and between ∠D2 and FTA (r = 0.273, p = 0.035).

The same analysis was performed separately for each sex, and in men, there was no significant correlation between FTA and ∠D1 (r = 0.216, p = 0.098), ∠D2 (r = 0.001, p = 0.997), and ∠D3 (r = -0.050, p = 0.702). In women, there were modest positive correlations between FTA and ∠D1 (r = 0.262, p = 0.043) and ∠D2 (r = 0.273, p = 0.035). There was no significant correlation between FTA and ∠D3 (r = 0.153, p = 0.242) (Figure 6).

## Discussion

In TKA, inadequate rotational alignment can lead to a variety of clinical and functional issues, including anterior knee pain, patellar instability, stiffness, reduced range of motion, and decreased patient satisfaction and functional scores [3,4]. Previously published reports on rotational alignment have demonstrated that Akagi’s line is a useful rotational reference AP axis for the proximal tibia, owing to its high consistency with the femoral AP axis [1]. However, there is no consensus regarding the distal AP axis. In this study, we evaluated the alignment consistency of 3 representative distal AP axes of the lower leg, namely, d-AP1: ankle AP axis, d-AP2: talar AP axis, and d-AP3: second metatarsal bone axis, with p-AP: Akagi’s line in patients with medial knee OA undergoing TKA. Our results showed that, when p-AP was set as the reference value, d-AP1 was -17.55° (p < 0.001), d-AP2 was -3.36° (p < 0.001), and d-AP3 was -0.83° (p = 0.406), with d-AP3 showing the closest agreement with the p-AP axis. Furthermore, this consistency remained stable regardless of the severity of OA.

The minimal angular deviation of d-AP3 from p-AP suggests that the second metatarsal bone axis can be used as a reliable supplemental reference for tibial rotational alignment, particularly in minimally invasive TKA procedures in which access to proximal tibial landmarks may be limited. Regarding the central position of the ankle joint, methods have been reported in which the center is positioned 3 to 5 mm medially from the midpoint between the medial and lateral malleoli [19,20], or in which the center is located at 57% lateral to the distance between the medial and lateral malleoli [21]. Additionally, although some reports indicate that the central position of the talus aligns with the lateral margin of the extensor hallucis longus tendon [22], intraoperative determination of the center of the ankle joint and talus is not straightforward. On the other hand, owing to its accessibility during surgery and its uniform properties among patients with various deformity severities and characteristics, d-AP3 can be considered to be a clinically useful reference axis.

As mentioned above, Akagi’s line has been established as a reliable reference for tibial rotation in normal knees, but few studies have rigorously evaluated distal reference axes using 3D imaging. The method of this study, using 3DCT for measuring the angle between p-AP and d-AP axes, improves measurement reproducibility and provides intuitive, anatomically relevant reference axes that can be readily visualized during surgery. This may facilitate more precise translation of preoperative measurements into intraoperative component placement. Previous reports have suggested a general agreement between distal and proximal AP axes [2,5-7], but some discrepancies can occur depending on the individual’s anatomy or deformity. Our study provides quantitative lines of evidence from 3DCT reconstructions that the second metatarsal bone axis most consistently aligns with p-AP among the three distal axes analyzed. In this study, measurements were obtained by two examiners, and the results demonstrated a high degree of reproducibility, with a maximum measurement error of 3°.

Interestingly, d-AP2 and d-AP3 showed statistically greater internal rotation in women than in men in this study, suggesting a potential sex-associated anatomical variation (Table 2) [23,24]. Specifically, d-AP3 was externally rotated by 1.3° in men, whereas it was internally rotated by 2.6° in women (p = 0.05). Similarly, d-AP2 was also significantly more internally rotated in women than in men (p = 0.006), whereas no significant sex difference was observed for d-AP1 (p = 0.16). Therefore, we should consider this variability when using d-AP2 or d-AP3 as the distal AP axis during surgery. However, d-AP3 was not significantly affected by the severity of deformity assessed using KL grade and FTA (Figures 3-5). In contrast, d-AP1 showed a significant correlation with FTA (Figure 4), suggesting that the ankle AP axis may rotate externally as FTA increases. This result supports the possibility that d-AP1 may not be a reliable indicator in patients with advanced varus deformity.

The results of this study suggest that while a certain trend exists in the relationship between p-AP and d-AP axes, there is also diversity primarily attributable to individual differences. Therefore, it is considered important to understand the relationship between p-AP and d-AP axes for each individual case during preoperative preparation.

When using the d-AP3 as an intraoperative reference axis, it is important to preoperatively assess the angular difference (∠D3) between the p-AP and d-AP axes using CT measurements. During tibial component trial placement, surgeons should ensure that this difference is appropriately reflected in the final rotational position of the component. This approach may help minimize rotational errors in total knee arthroplasty.

This study has several limitations. This was a retrospective, single-center analysis with a relatively small sample size. However, small to moderate effect sizes were obtained for all significant comparisons. The study population was limited to Japanese patients. Therefore, the results may reflect typical Asian characteristics, and different anatomical features may exist in other ethnic groups [20]. Additionally, this study was limited to patients with medial knee OA, and generalizing the findings to lateral knee OA, post-traumatic knee OA, and RA is challenging. In other words, axis relationships may differ in non-medial-type knees. Potential measurement bias may have existed in the CT-based measurements of this study. Variations in patient positioning during imaging, in particular, could be a source of measurement bias. Although braces were used to standardize knee and ankle angles in this study, some patients had limited knee extension, potentially making imaging position bias unavoidable. Only one of the proximal AP axes used in TKA was analyzed in this study. If other axes were considered as the proximal tibial AP axis, the results may differ. However, at present, Akagi’s line is recognized as the gold standard for the proximal AP axis in TKA and has high generalizability. Finally, the measurement results are based on static 3DCT images and have not been evaluated for intraoperative reproducibility or postoperative clinical outcomes. Further studies are needed to evaluate the intraoperative applicability of d-AP3, particularly in navigation-assisted or robot-assisted TKA. Future prospective studies correlating intraoperative alignment using d-AP3 with clinical outcomes, such as knee joint range of motion and changes in patients' activities of daily living, are expected to clarify the utility of d-AP3 in TKA.

## Conclusions

Of the three d-AP axes investigated in this study, d-AP3 (second metatarsal bone axis) showed the highest degree of alignment with the p-AP axis (Akagi’s line) and was found to be least affected by varus deformity with progression of medial knee OA. Although variabilities owing to anatomical differences in the distal ankle region associated with sex differences should be noted, d-AP3 was suggested to be an easily identified and reliable reference axis for tibial rotational alignment during TKA surgery.
